# Current treatment for diabetes: a holistic approach

**DOI:** 10.1007/s42000-025-00734-3

**Published:** 2025-11-26

**Authors:** Niki G. Mourelatou, Dimitris Kounatidis, Natalia G. Vallianou, Giuseppe Daniele, Angela Dardano, Eleni Rebelos

**Affiliations:** 1 Second Department of Internal Medicine, NIMTS Hospital, Athens, 11521 Greece; 2 Diabetes Center, First Department of Propaedeutic and Internal Medicine, Medical School, National and Kapodistrian University of Athens, Laiko General Hospital, Athens, 11527 Greece; 3 First Department of Internal Medicine, Sismanogleio General Hospital, Athens, 15126 Greece; 4 Department of Clinical and Experimental Medicine, University of Pisa, Pisa, 56126 Italy

**Keywords:** Diet, Exercise, Glucose-lowering drugs, Latent autoimmune diabetes in adults (LADA), Maturity-onset diabetes of the young (MODY), Secondary diabetes, Type 1 diabetes, Type 2 diabetes

## Abstract

In recent years, the global prevalence of diabetes has increased significantly, with type 2 diabetes (T2D) being recognized as a major public health concern. Its intricate and heterogeneous pathophysiology, the potential for serious complications, and the strong influence of lifestyle factors on glycemic control underscore the need for individualized treatment strategies. Effective management should integrate both tailored pharmacotherapy and lifestyle interventions, adapted to each patient’s unique needs. The aim of this narrative review is to explore the essential components of optimal diabetes care, emphasizing the importance of personalized approaches. We highlight findings from key cardiovascular outcome trials (CVOTs) to demonstrate the benefits of certain therapeutic agents that extend beyond glycemic control. Finally, drawing from our clinical experience, we propose practical strategies for the comprehensive, patient-centered management of diabetes.

## Introduction

 Diabetes comprises a heterogeneous group of disorders characterized by a chronic increase of plasma glucose levels due to inadequate insulin secretion from pancreatic β-cells. The extent of β-cell dysfunction varies across diabetes types. In type 1 diabetes (T1D), β-cell failure is absolute and results from autoimmune destruction; by the time of diagnosis, most β-cells have already been lost. In contrast, type 2 diabetes (T2D) is characterized by relative β-cell dysfunction. During the early stages of T2D, insulin secretion may be normal or even elevated, but it is insufficient to overcome insulin resistance (IR) in key metabolic tissues such as skeletal muscle, adipose tissue, and the liver [1].

The rising prevalence of obesity has also impacted individuals with T1D, leading to a new subset of diabetes termed “double diabetes,” which refers to the coexistence of autoimmune diabetes and IR [2]. Between the two main types lies a spectrum of other diabetes forms. Latent autoimmune diabetes in adults (LADA) presents with diabetes-associated autoantibodies but has a slower progression and develops in adulthood; its severity depends on the degree of β-cell destruction. On the other hand, maturity-onset diabetes of the young (MODY) is a monogenic form of diabetes with autosomal dominant inheritance, typically presenting with mild hyperglycemia and in patients with a strong family history [3]. Additional secondary forms of diabetes include pancreatogenic or type 3c diabetes (T3cD) and drug-induced diabetes, both of which are often challenging to manage and frequently require intensive insulin therapy [3].

Over the past several decades, the incidence of diabetes has steadily increased. T2D, in particular, is recognized as a significant global health burden [4]. In 2024, an estimated 589 million adults, roughly 1 in 9 people worldwide, were living with diabetes, a figure projected to rise to 853 million by 2050. T2D currently accounts for one death every 6 s, with healthcare costs exceeding one trillion U.S. dollars annually [5]. Furthermore, diabetes-related complications, including cardiovascular disease (CVD), chronic kidney disease (CKD), lower limb amputations, and blindness, contribute to increased morbidity and mortality, while significantly affecting patients’ quality of life (QoL) [6].

Given the multifaceted nature of diabetes and its associated complications, a one-size-fits-all approach to treatment is no longer considered adequate. Meanwhile, the evolving landscape of pharmacological therapies, combined with growing recognition of the role of lifestyle and social determinants of health, necessitates a more individualized treatment approach. This narrative review aims to examine the key pillars of contemporary diabetes management, with a focus on evidence-based therapeutic choices, the role of cardiovascular outcome trials (CVOTs) in guiding clinical decisions, and the practical application of personalized care in everyday practice. Finally, recognizing the central role of personalized treatment in diabetes care, we share practical insights and reflections on general management strategies informed by our clinical experience.

## Lifestyle modification

### Dietary interventions

Addressing excess body weight is a pivotal therapeutic objective in the management of T2D. Even modest weight loss (3–7%) can significantly improve glycemic control and reduce cardiovascular (CV) risk factors, while weight reduction exceeding 10% may lead to remission of T2D and confer additional benefits for associated metabolic conditions [7]. Nutrition and physical activity, as modifiable lifestyle factors, are central to both the prevention and management of T2D. When combined with behavioral interventions, they can further enhance weight loss efforts and metabolic outcomes [8]. Extensive research has evaluated various dietary approaches to identify the most effective regimen; however, it is now well recognized that no universal “ideal” diet exists [9].

As emphasized by the American Diabetes Association (ADA) Standards of Care, nutritional strategies must be individualized. Consequently, the focus has shifted from isolated macronutrient and micronutrient targets to comprehensive, individualized medical nutrition therapy (MNT) tailored to each patient’s cultural preferences, lifestyle, and health status [10]. A reduction in glycated hemoglobin (HbA_1c_) of up to 2% may be observed when there is regular support from a qualified dietitian or nutritionist [11]. A pooled analysis of four randomized controlled trials (RCTs) published in 2023 by Sathish et al. showed that conventional lifestyle interventions reduce the risk of T2D in individuals with impaired glucose tolerance, with or without impaired fasting glucose, but not in those with isolated impaired fasting glucose [12]. Similarly, the Diabetes Prevention Program (DPP) demonstrated that lifestyle interventions were significantly more effective than metformin in delaying T2D onset in high-risk individuals [13]. Interestingly, a meta-analysis of 63 studies in 2018 demonstrated that high-risk individuals receiving lifestyle interventions targeting weight loss reduced the risk of T2D by 29%, even with limited weight reduction [14].

Several dietary patterns are considered appropriate for individuals with T2D, including the Mediterranean diet, Dietary Approaches to Stop Hypertension (DASH), low-fat diets, carbohydrate-restricted diets, vegetarian diets, and vegan diets [9]. The Mediterranean diet is frequently recommended due to its emphasis on monounsaturated and polyunsaturated fats, long-chain fatty acids, and high consumption of whole, plant-based foods, all of which are associated with improved glycemic control and lipid profiles and reduced CV risk [15]. Core components include fresh fruits and vegetables, whole grains, legumes, nuts, and olive oil as the primary fat source, while intake of added sugars, sugary beverages, sodium, alcohol, processed foods, refined carbohydrates, and saturated fats is minimal [16]. A simplified and practical visual guide is the Diabetes Plate Method, which allocates half the plate to non-starchy vegetables, one quarter to lean proteins, and one quarter to carbohydrates [17]. Adherence to diets such as the Mediterranean and DASH dietary patterns is associated with reduced risk of T2D, while diets high in glycemic index/load, red or processed meats, and sugar-sweetened beverages increase this risk [18]. A meta-analysis by Papamichou et al. in 2019, which included 20 RCTs lasting more than 6 months, found that Mediterranean, vegetarian, and vegan diets improved glycemic control in patients with T2D. However, further research is required to evaluate their long-term sustainability and effectiveness [19]. The ketogenic diet has shown similar reductions in HbA_1c_ compared to the Mediterranean diet over a 12-week period but has been linked to elevated low-density cholesterol (LDL-C) and lower intakes of certain essential nutrients [20]. Intermittent fasting may offer benefits in individuals with T2D, although evidence remains limited for those with T1D [21].

Supplement use is generally not recommended in the absence of documented deficiencies [9]. Notably, vitamin D supplementation, although widely studied, is not currently advised without confirmed deficiency due to inconsistent evidence regarding its impact on diabetes outcomes [22–24]. Individuals taking metformin should undergo periodic monitoring of vitamin B12 levels every 2 to 3 years, especially in the presence of anemia or neuropathy, and multivitamin supplements may be appropriate for certain populations, such as pregnant women or those on vegetarian/vegan diets [25]. Food and Drug Administration (FDA)-approved non-nutritive sweeteners (NNS) may serve as alternatives to sugar but appear to have neutral effects on glycemic control [26].

### Physical activity

Physical activity is equally vital in diabetes care. It improves glycemic control, supports weight management, and enhances overall health: it should be individualized to each patient’s abilities and preferences [27]. Regardless of the type, exercise enhances glucose uptake in skeletal muscle via insulin-independent pathways, thereby improving insulin sensitivity [28]. Current guidelines recommend at least 150 min of moderate-intensity aerobic activity per week spread over 3 or more days, along with 2–3 sessions of resistance training [25]. Even short-term aerobic exercise can enhance glycemic control by improving insulin sensitivity and reducing hepatic glucose production [29], while the combination of aerobic and resistance training is associated with greater HbA_1c_ reductions [30]. Interestingly, activities promoting flexibility and balance, such as yoga, may be especially useful in older adults [25].

A meta-analysis of 14 studies confirmed that physical activity lowers HbA_1c_ levels independently of weight changes, thereby reducing the risk of complications [31]. High-intensity interval training (HIIT) may benefit both T2D and T1D patients by enhancing glycemic control, reducing insulin requirements, and improving cardiometabolic parameters [32]. In T1D, however, exercise must be approached cautiously due to the risks of hypoglycemia [33].

Routine cardiac screening before initiating exercise is not universally recommended, but a thorough medical history is essential [34]. Patients with complications may require specific precautions, namely, those with proliferative or severe non-proliferative retinopathy should avoid high-intensity activity due to risks of retinal detachment or hemorrhage, while patients with autonomic neuropathy may need a CV evaluation before intense exercise [28]. Individuals with peripheral neuropathy should wear protective footwear and inspect their feet regularly, whereas no specific restrictions apply to those with CKD [25]. Physical activity is especially important in the context of sarcopenia and sarcopenic obesity, which are increasingly common in diabetes and may be exacerbated by certain antidiabetic medications [35].

### Behavioral therapy and psychological support

Behavioral therapy and psychological support are integral to effective diabetes management [36]. Psychological disorders such as depression and anxiety are common among individuals with diabetes, and the burden of self-care can contribute to a condition known as diabetes distress [36, 37]. This can negatively affect adherence, glycemic control, and QoL [38]. Evidence indicates that nearly 20% of people with T1D experience high diabetes-related distress [39], while rates in T2D may reach 60% [40]. Support from behavioral health professionals with expertise in diabetes can be highly beneficial, integrating education, psychosocial interventions, and digital tools to enhance care [10]. An RCT demonstrated that mindful self-compassion interventions led to significant HbA_1c_ reductions [41], whereas cognitive behavioral therapy (CBT) has been shown to alleviate distress, anxiety, and depression, facilitating better adherence to dietary and physical activity goals [42]. Identifying eating disorders is also essential, as they are closely tied to diabetes outcomes [43].

Overall, a successful lifestyle intervention promotes a balanced, nutrient-dense diet in appropriate portions, regular physical activity, and sustainable behavior change. These strategies aim to improve glycemic control, support weight loss, manage blood pressure and lipid levels, and enhance overall health. Patient education is fundamental to encouraging long-term adherence. When lifestyle interventions are insufficient to achieve clinical goals, adjunctive pharmacotherapy or metabolic bariatric surgery (MBS) should be considered [10]. While MBS is a valuable option for selected patients, it carries inherent risks [44, 45] and often results in more modest weight loss among individuals with T2D compared to those with severe obesity alone [46].

## Timeline of diabetes drug development and the “boost” of rosiglitazone in diabetes management

Insulin, the first pharmacological treatment for diabetes, was discovered in 1921 by Sir Frederick Banting, Charles Best, and J.J.R. Macleod at the University of Toronto, Canada. This groundbreaking achievement earned a Nobel Prize and, more importantly, transformed T1D from a fatal disease into a manageable chronic condition. Prior to the availability of insulin, individuals diagnosed with T1D rarely survived more than 2 to 5 years post-diagnosis. Although metformin was also discovered in the 1920 s, its use as a glucose-lowering drug was not approved until 1957 [47]. Around the same time, sulfonylureas (SUs) were introduced, becoming the mainstay of oral pharmacotherapy for T2D throughout the mid-20th century [48]. For several decades, diabetes management relied heavily on these three agents, which remained the cornerstone of treatment.

A turning point came in the late 1990 s with the introduction of additional drug classes, including thiazolidinediones (TZDs) and α-glucosidase inhibitors. These new therapies expanded the armamentarium for glycemic control and targeted different mechanisms of action in diabetes pathophysiology. However, the true paradigm shift in diabetes pharmacotherapy began in the early 2000 s and gained momentum in the following decades with the introduction of incretin-based therapies and sodium-glucose co-transporter-2 inhibitors (SGLT2is). The recognition of incretin dysfunction in diabetes marked a key point in the development of novel antidiabetic agents. Incretin-based therapies appeared in the mid-2000s and include the following three main classes: dipeptidyl peptidase-4 inhibitors (DPP-4is), glucagon-like peptide-1 receptor agonists (GLP-1 RAs), and, lately, tirzepatide, a dual GLP-1/*g*lucose-dependent insulinotropic polypeptide receptor agonist (GLP-1/GIP RA). The therapeutic landscape of diabetes further expanded with the approval of the first SGLT2i, canagliflozin, in March 2013. Among these newer drug classes, GLP-1 RAs and SGLT2is offer significant CV and renal benefits [47].

A pivotal event that influenced the course of diabetes drug development was the controversy surrounding the thiazolidinedione rosiglitazone. Initially introduced as a potent insulin sensitizer, rosiglitazone gained rapid popularity due to its efficacy in reducing IR and improving glycemic parameters. However, in 2007, a widely publicized meta-analysis suggested an increased risk of CV events associated with its use. Although the analysis was later criticized for methodological limitations, the concerns it raised prompted a major shift in regulatory policy [49]. In response, the U.S. FDA issued guidance in 2008 requiring that all new glucose-lowering therapies demonstrate CV safety prior to approval. Specifically, the FDA mandated that drug sponsors conduct large-scale CVOTs to confirm that their products did not increase the risk of major adverse cardiovascular events (MACE), including CV death, nonfatal myocardial infarction, and nonfatal stroke. This regulatory milestone effectively redefined the standards for diabetes drug development, prioritizing not only glycemic efficacy but also long-term safety and organ protection [50]. Since then, all newly approved antidiabetic medications have been required to undergo rigorous CVOTs, marking the beginning of a new era in diabetes care that emphasizes individualized treatment strategies with both metabolic and CV benefits.

## Drug therapy in type 2 diabetes

### Metformin

For decades, metformin has been the cornerstone of pharmacotherapy in patients with T2D. The landmark U.K. Prospective Diabetes Study (UKPDS), published in 1998, demonstrated metformin’s ability not only to lower blood glucose but also to mitigate diabetes-related mortality [50]. Its glucose-lowering effect is primarily due to the inhibition of hepatic gluconeogenesis, thereby improving insulin sensitivity [51]. Metformin is thought to act via inhibition of mitochondrial respiratory chain complex I and activation of AMP-activated protein kinase (AMPK), both of which modulate energy metabolism and suppress hepatic glucose production [52].

The drug is highly effective, although gastrointestinal side effects are common. To improve tolerability, slow dose escalation, the use of extended-release formulations, and administration with food are suggested. In particular, metformin should be initiated at a low dose, typically 500 to 850 mg once daily, and titrated gradually by increasing the dose every 1 to 2 weeks up to the maximum tolerated dose. While the recommended upper limit is 3 g per day, a daily dose of 2 g is usually sufficient, as higher doses offer limited additional benefit. Dose adjustments are necessary in patients with renal impairment due to the risk of lactic acidosis, and the drug should be discontinued if estimated glomerular filtration rate (eGFR) falls below 30 mL/min/1.73 m^2^. According to the ADA and European Association for the Study of Diabetes (EASD) guidelines, initial combination therapy may be considered rather than starting with monotherapy, depending on baseline HbA_1c_ and patient-specific characteristics [53].

### Sulfonylureas

SUs, which stimulate insulin secretion by binding to the SU receptor subunit of ATP-sensitive potassium channels in pancreatic β-cells, were once a mainstay of therapy alongside metformin [54]. They are classified into three generations, with second- and third-generation agents being the most commonly prescribed. However, their insulinotropic effect is glucose-independent, which significantly increases the risk of hypoglycemia. SUs may additionally exert metabolic stress on β-cells, potentially accelerating β-cell exhaustion and hastening the need for insulin therapy [55]. Moreover, concerns about their CV safety exist, with data suggesting a possible association with increased CV risk [56]. Given these safety issues, the use of SUs, especially in older adults, has markedly declined. Nonetheless, clinical inertia may explain their continued prescription in certain settings. Even in patients with stable glycemic control, their use should generally be avoided due to the persistent risk of severe hypoglycemia, particularly in patients with CKD [48]. Despite these considerations, SUs remain relevant in resource-limited settings due to their low cost [48] and are particularly beneficial in patients with MODY3 and MODY1 subtypes where they outperform metformin [57].

### Thiazolidinediones

TZDs represent a distinct class of antidiabetic agents that act through the activation of the peroxisome proliferator-activated receptor gamma (PPAR-γ). This leads to improved insulin sensitivity, resulting in potent, albeit delayed, antihyperglycemic effects. Three major TZDs have been marketed to date, namely, troglitazone, rosiglitazone, and pioglitazone, all derived from the prototype compound ciglitazone. Troglitazone was withdrawn due to rare but severe cases of acute liver failure, while rosiglitazone was later removed from the market amid concerns about CV safety, as previously discussed. Currently, pioglitazone is the only major TZD still in use in Europe, though its application is limited due to adverse effects, including fluid retention, exacerbation of heart failure (HF), weight gain, and an increased risk of bone fractures [58]. In addition, pioglitazone should be avoided in patients with a history of bladder cancer as it has been associated with an elevated risk of this malignancy, although more recent studies have challenged the strength of this link [59]. The safety concerns associated with TZDs have prompted the development of newer agents with more selective or combined actions. These include partial or selective PPAR-γ agonists, dual PPAR-α/γ agonists, such as saroglitazar, and pan-PPAR agonists, such as lanifibranor, which have shown promising effects on various parameters of metabolic health, including T2D and its comorbidities. However, these findings remain preliminary and require further validation in larger trials [60, 61].

### Incretin-based therapies

Recognition and therapeutic targeting of the incretin effect has marked a major milestone in the management of T2D. The incretin phenomenon refers to the observation that oral glucose intake elicits a greater insulin response than intravenous glucose administration due to the action of gut-derived hormones GLP-1 and GIP. In individuals with T2D, this response is blunted, contributing to impaired insulin secretion and dysregulated glucose control. Therapeutic targeting of the incretin pathway involves the following three main classes of drugs: DPP-4is, GLP-1 RAs, and dual GLP-1/GIP RAs.

#### Dipeptidyl peptidase-4 inhibitors

DPP-4is act upstream on the incretin hormone axis by inhibiting the enzyme dipeptidyl peptidase-4 (DPP-4). This enzyme is responsible for the rapid degradation of endogenous GLP-1 and GIP. By blocking DPP-4, these agents prolong the activity of incretin hormones, modestly enhancing insulin secretion and suppressing glucagon in a glucose-dependent manner. DPP-4is were once commonly used in combination with metformin. However, due to their moderate glycemic efficacy and neutral impact on weight, CV, and renal outcomes, their use has diminished over time. Still, they may be appropriate for patients with mild hyperglycemia, particularly those reluctant to escalate therapy or in healthcare settings with limited access to newer agents [62]. In addition, DPP-4is remain a reasonable option for certain older adults at risk of hypoglycemia, as their safety profile is generally favorable, with adverse effects typically being mild and infrequent. All DPP-4is require dose adjustment in renal impairment, except for linagliptin, which is particularly suitable for individuals with advanced CKD. Linagliptin is also available in single-pill combinations with SGLT2is. Importantly, DPP-4is should not be used concurrently with GLP-1 RAs, due to their overlapping mechanisms of action and lack of additive benefit [63].

#### Glucagon-like peptide 1 receptor agonists

GLP-1 RAs mimic endogenous GLP-1, enhancing glucose-dependent insulin secretion, suppressing glucagon, delaying gastric emptying, and promoting satiety and β-cell preservation [64]. Earlier agents (e.g., liraglutide and exenatide) have largely been replaced by once-weekly formulations such as dulaglutide and semaglutide, which offer superior convenience and efficacy. An oral formulation of semaglutide is also available and can be used especially in patients who do not wish to start an injectable agent (for instance, in patients with needle phobia). This formulation has similar effects as other GLP-1RA on diabetes control, weight loss, and CV outcomes, but it requires a specific mode of administration at least 30 min before taking other drugs or eating [65, 66].

CV benefits of GLP-1 RAs, particularly for liraglutide, were first shown in the LEADER (Evaluation of Cardiovascular Outcome Results) trial in 2016, where CV mortality and MACE were significantly reduced [67]. Similar findings were reported with semaglutide in the SUSTAIN-6 (Trial to Evaluate Cardiovascular and Other Long-term Outcomes with Semaglutide in Subjects with Type 2 Diabetes) in 2016 [68], and with dulaglutide in the REWIND (Researching Cardiovascular Events with a Weekly Incretin in Diabetes) trial in 2019, which included a broader population at lower CV risk [69]. Recently, in 2024, the FLOW (Evaluate Renal Function with Semaglutide Once Weekly) study presented a new therapeutic role of GLP-1RA in managing CKD progression among T2D patients. Specifically, the risk of a primary composite outcome, including renal outcomes, onset of kidney failure, at least a 50% decline in eGFR from baseline, renal or CV death, was 24% lower in the semaglutide group than in the placebo group [70].

Dual GLP-1/GIP RAs, particularly tirzepatide, have recently been integrated into diabetes management guidelines, offering a novel therapeutic approach. Their full effects on MACE, HF, and CKD are currently under investigation [53]. The SURPASS (a Study of the Efficacy and Safety of Tirzepatide) clinical trial program has demonstrated the superiority of tirzepatide over placebo and several established glucose-lowering agents in terms of both glycemic control and weight loss [71]. Notably, the SURPASS-2 trial (Tirzepatide versus Semaglutide Once Weekly in Patients with Type 2 Diabetes), published in 2021, compared tirzepatide to semaglutide and found that, after 40 weeks, participants receiving tirzepatide achieved greater reductions in HbA_1c_ — 2.01%, 2.24%, and 2.30% with the 5 mg, 10 mg, and 15 mg doses, respectively — compared to a 1.86% reduction in those treated with semaglutide 1 mg [72].

The above findings provide strong support for the use of GLP-1 RAs and dual GLP-1/GIP RAs as effective second-line, and in selected patients, first-line, therapies in the management of T2D. Beyond their robust glucose-lowering effects, these agents offer marked CV and potential renal protection. Importantly, they also enable substantial and sustained weight loss, making them particularly suitable for subjects with T2D who are overweight or obese. Their prioritization in such populations is strongly advocated.

### Sodium-glucose cotransporter-2 inhibitors

SGLT2is act by blocking the SGLT2 protein located on the luminal surface of the distal segment of the proximal convoluted tubule (PCT), promoting urinary glucose excretion. Given their site of action, adequate glomerular filtration is essential for SGLT2is to reach the PCT. As such, their glucose-lowering efficacy is moderate to high but becomes minimal when eGFR falls below 45 ml/min/1.73 m^2^. Their prescription should be carefully considered in individuals prone to genital mycotic infections, while all patients should be educated on optimal genital hygiene to minimize adverse effects and reduce the likelihood of treatment discontinuation. Furthermore, these agents have been associated with an increased risk of euglycemic diabetic ketoacidosis (DKA), although this complication is rare. The three main agents in this drug class are empagliflozin, dapagliflozin, and canagliflozin.

SGLT2is have ushered in a new era in the management of T2D. The landmark EMPA-REG OUTCOME (Empagliflozin Cardiovascular Outcome Event Trial in Type 2 Diabetes Mellitus Patients-Removing Excess Glucose), published in 2015, demonstrated that adding empagliflozin to standard care in patients with T2D and high CV risk significantly reduced CV mortality by 38%, HF hospitalizations by 35%, and all-cause mortality by 32% over a median of 2.6 years [73]. These findings were particularly notable, as prior studies had failed to show any CV benefit from glucose-lowering drugs in patients with HF [74, 75]. It soon became clear that these benefits were class effects. In the CANVAS (Canagliflozin Cardiovascular Assessment Study, published in 2017), and the DECLARE-TIMI 58 (Dapagliflozin Effect on Cardiovascular Events-Thrombolysis in Myocardial Infarction 58, published in 2019), canagliflozin and dapagliflozin, respectively, showed favorable outcomes on HF and CV endpoints in patients with varying degrees of CV risk [76, 77].

A key innovation in the use of SGLT2is is their ability to deliver CV and renal benefits regardless of diabetes status. Supported by high-quality clinical trials, this has expanded their therapeutic role beyond glycemic control to include treatment for HF and CKD [78]. The DAPA-HF (Dapagliflozin And Prevention of Adverse outcomes in Heart Failure) study, in 2019, was pivotal in this regard, showing that dapagliflozin significantly reduced the risk of worsening HF or CV death in patients with heart failure with reduced ejection fraction (HFrEF), regardless of whether they had diabetes [79]. Subsequent studies, such as the EMPEROR-Preserved (EMPagliflozin outcomE tRial in Patients With chrOnic heaRt Failure With Preserved Ejection Fraction), published in 2021, and DELIVER (Dapagliflozin Evaluation to Improve the Lives of Patients with Preserved Ejection Fraction Heart Failure), published in 2022, further extended the benefits of empagliflozin and dapagliflozin to patients with heart failure with preserved ejection fraction (HFpEF) [80, 81].

On the renal side, in 2019, the CREDENCE (Canagliflozin and Renal Events in Diabetes with Established Nephropathy Clinical Evaluation) trial was the first to show that canagliflozin, added to standard therapy with angiotensin-converting enzyme (ACE) or angiotensin receptor blockers (ARBs), significantly reduced the risk of end-stage renal disease (ESRD), doubling of serum creatinine, and renal or CV death in patients with T2D and CKD. Importantly, nearly half of the study population did not have established CVD, making CREDENCE the first trial to show renal and MACE benefits in the context of primary prevention [82]. Following this, in 2020, the DAPA-CKD (Dapagliflozin and Prevention of Adverse Outcomes in Chronic Kidney Disease) trial demonstrated that in patients with CKD, those treated with dapagliflozin had a lower risk of sustained decline in eGFR of at least 50%, progression to ESRD, or death due to renal or CV reasons, compared to the placebo group, whether or not they had T2D [83]. In 2022, the EMPA-KIDNEY (Study of Heart and Kidney Protection with Empagliflozin) confirmed that empagliflozin reduced the risk of CKD progression and CV death across a broad spectrum of CKD, again independent of diabetes status [84]. In fact, empagliflozin reduced cardiorenal events by nearly 28% and benefits persisted even 1 year after discontinuation [85, 86].

In summary, SGLT2is are now considered mainstay treatments for patients with T2D due to their robust effects on HF prevention, reduction of albuminuria, and slowing of CKD progression. Notably, in individuals with established CVD or those at high CV risk, such as patients aged ≥ 55 years with multiple risk factors (e.g., obesity, hypertension, dyslipidemia, albuminuria, or smoking), SGLT2is may be prescribed as first-line agents, even prior to metformin, as part of a personalized therapeutic strategy. Regarding atherosclerotic outcomes, empagliflozin and canagliflozin showed the most consistent reductions in MACE. At the same time, large RCTs support the use of dapagliflozin and empagliflozin in patients with heart failure, both with reduced and preserved ejection fraction. Renal benefits have been demonstrated with canagliflozin, dapagliflozin, and empagliflozin in their respective landmark trials. Nonetheless, head-to-head studies are still needed to better define potential differences among agents within this pharmacological class.

### Second-generation longer-acting basal insulins and once-weekly insulin therapy

As T2D is a progressive disease, many patients eventually require insulin therapy when glycemic targets are no longer achieved with combinations of non-insulin glucose-lowering agents. However, insulin initiation is often delayed due to patient reluctance and clinical inertia, even among healthcare professionals, despite suboptimal glycemic control on maximal oral or injectable therapy. In certain cases, such as individuals with newly diagnosed T2D and HbA_1c_ ≥ 10%, basal insulin may be necessary early in the disease course to achieve prompt glycemic control. While basal-bolus insulin regimens are rarely used in early-stage T2D today, they may still be required in advanced disease when all other treatment options fail to maintain acceptable glycemic levels [87].

Second-generation basal insulin analogs, such as insulin degludec and insulin glargine U300, offer flatter and more stable pharmacokinetic and pharmacodynamic profiles compared to insulin glargine U100. These newer formulations are associated with a lower risk of hypoglycemia, particularly nocturnal episodes [88, 89]. Head-to-head comparisons have produced varied findings. In insulin-naïve patients, the BRIGHT (Digital Therapeutic Based Investigation to Improve Glycemic Control in Patients With Type 2 Diabetes and Residual Hyperglycemia on Stable Medical Therapy) study, in 2018, demonstrated similar glycemic control with both agents but fewer events of hypoglycemia during the titration phase with glargine U300 [90]. Conversely, in 2020, the CONCLUDE (Comparison of the Once-daily Novel insulin glargine 300 U/mL versus insulin DeglUdec in type 2 Diabetes patients with hypoglyCaEmia risk) trial, which involved patients with prior insulin use, showed lower rates of nocturnal symptomatic and severe hypoglycemia with degludec compared to glargine U300 [91]. Overall, the two insulins are comparable in terms of efficacy and safety, with the choice between them often guided by practical considerations, such as cost and availability [90]. Current guidelines recommend initiating basal insulin at either a fixed dose of 10 units daily or based on body weight (0.1–0.2 units/kg/day). Titration should continue until fasting plasma glucose (FPG) reaches target levels (typically 90–130 mg/dL). A commonly suggested adjustment is an increase of two units every 3–4 days, though titration may be personalized. If fasting hypoglycemia occurs, the basal insulin dose should be reduced immediately [92].

Recently, once-weekly basal insulin analogs have been developed, offering the potential for improved adherence and reduced treatment burden. Insulin icodec is the first such analog to become available, although its use is currently limited to a few countries. In 2023, the randomized, double-blind ONWARDS 3 (Once-Weekly Insulin Icodec Versus Once-Daily Degludec in Adults With Type 2 Diabetes) study compared icodec to degludec in insulin-naïve patients with T2D over 26 weeks. The study demonstrated that icodec was both noninferior and superior to degludec in HbA_1c_ reduction, with no significant differences in weight change. Although the incidence of episodes of hypoglycemia was higher with icodec, the overall rates remained low in both groups. Until today icodec is the only available once-weekly insulin, although it is still available in only a few countries [93]. Another once-weekly insulin formulation, efsitora, was evaluated in the QWINT-2 (Once Weekly Insulin Therapy) trial in 2025. In this study of insulin-naïve patients, once-weekly efsitora was found to be noninferior to once-daily degludec in terms of HbA_1c_ reduction, with no significant differences in hypoglycemia rates between groups [94]. The introduction of once-weekly insulin therapies may significantly improve patient acceptance and adherence, particularly among those hesitant to initiate insulin. This potential is supported by prior experience with once-weekly GLP-1 RAs, which demonstrated better treatment adherence compared to their once-daily counterparts [95]. Such advancements could also help overcome treatment delays and facilitate timely insulin initiation in appropriate patients.

## Drug therapy in type 1 diabetes

The cornerstone of treatment for T1D is intensive insulin therapy, delivered either through multiple daily injections (MDIs) or continuous subcutaneous insulin infusion (CSII) via an insulin pump. In parallel, ongoing research aims to identify therapies that may help preserve residual β-cell function and slow autoimmune destruction in early T1D. Patients with T1D typically require 30–50% of their total daily insulin as basal insulin, with the remaining percentage allocated to prandial needs [53]. Accurate carbohydrate counting is essential for optimal postprandial glucose control, and patients must be educated early in their treatment journey to adjust mealtime insulin accordingly. The insulin-to-carbohydrate ratio (ICR) estimates the number of insulin units needed per gram of carbohydrate consumed, while the insulin sensitivity factor (ISF) represents the expected decrease in blood glucose (mg/dL) from 1 unit of rapid-acting or ultra-rapid-acting insulin [96].

Because insulin therapy is lifelong, avoiding injection into areas with lipohypertrophy is critical for consistent absorption, although it can be challenging in practice. Continuous glucose monitoring (CGM) systems have been shown not only to improve glycemic control, but also to enhance QoL and reduce the risk of hypoglycemia, and their use is strongly recommended for all patients with T1D [53, 97]. The use of CSII further improves glycemic outcomes and should be considered in all patients capable of managing the device safely. With the advent of automated insulin delivery (AID) systems, patients primarily need to announce meals and input carbohydrate content and adjust for high-fat meals, while the algorithm automatically manages basal and correction dosing in real time [53].

While SGLT2is are not approved for T1D management due to the increased risk of DKA [98], they are occasionally prescribed off-label in highly selected, well-controlled patients to leverage their glycemic and cardiorenal benefits, but only under close clinical supervision. Similarly, metformin may be used in patients with T1D who exhibit features of insulin resistance (e.g., obesity and high insulin requirements). Recent RCTs have also evaluated the use of GLP-1RAs as adjunctive therapy in T1D. Results showed modest reductions in HbA_1c_, body weight, and total insulin dose, particularly in overweight or obese patients, although these agents are not yet approved for use in T1D [99].

Lastly, since autoimmunity is the central pathophysiological mechanism in T1D, research has increasingly focused on developing immune-targeted therapies aimed at preventing or delaying the loss of functional β-cells by targeting autoreactive T cells and modulating systemic immune dysregulation. Treatments such as anti-CD3 antibodies (e.g., teplizumab) and B cell-directed therapies have demonstrated some success in preserving β-cell function. However, challenges remain in balancing therapeutic efficacy with potential risks, including immune suppression [100].

## Drug therapy in other forms of diabetes

### Latent autoimmune diabetes in adults (LADA)

Although LADA is not officially recognized as a separate diabetes type and is generally considered a form of T1D, its management requires a distinct and individualized approach [101]. Representing approximately 2–12% of all diabetes cases, LADA is often misdiagnosed due to its heterogeneous clinical presentation [102]. Post-diagnosis, treatment should be personalized, with a dual goal of preserving β-cell function and achieving optimal metabolic control [103]. Although formal guidelines are lacking, therapeutic decisions are often guided by C-peptide levels: values below 0.3 nmol/L indicate a clinical profile similar to T1D requiring insulin treatment, while levels above 0.7 nmol/L support an approach similar to that for T2D, with individualized adjustments and regular monitoring; intermediate values necessitate ongoing reassessment [104]. Lifestyle modifications are encouraged from diagnosis, and oral hypoglycemic agents may be initiated, excluding those that may accelerate β-cell decline, like SUs. Over time, most patients will require insulin [104]. Among non-insulin agents, metformin, TZDs, GLP-1 RAs, SGLT2is, and DPP-4is have all been used to improve glycemic control and reduce complications [104, 105]. Notably, combining insulin with DPP-4is or using TZDs may be beneficial, while newer agents warrant further investigation [105]. DPP-4is, particularly in combination with vitamin D, have shown potential immunomodulatory effects, which is of interest given the autoimmune basis of LADA. Immunomodulatory therapies are under investigation but remain experimental [106, 107].

### Monogenic forms of diabetes

The main forms of monogenic diabetes are neonatal diabetes and MODY. Neonatal diabetes is diagnosed within the first 6 months of life and may be transient or permanent. It is often due to mutations in *KCNJ11* or *ABCC8* genes encoding the Kir6.2 and SUR1 subunits of the pancreatic β-cell ATP-sensitive potassium channel. Thus, many patients can be effectively treated with SUs instead of insulin [108].

MODY accounts for about 1% of all diabetes cases. It comprises at least 14 subtypes, each caused by a mutation in a single gene and inherited in an autosomal dominant pattern [109]. Despite its non-autoimmune, non-insulin-dependent nature, MODY is frequently misdiagnosed as either T1D or T2D [110], while MODY subtypes 1 to 3 make up the majority of cases, accounting for ~ 95% of all cases [111]. MODY 2 is caused by mutations in the glucokinase (GCK) gene; it leads to mild, stable hyperglycemia and often does not require treatment, except during pregnancy, when insulin may be necessary [111, 112]. On the other hand, MODY 1 and 3, caused by mutations in the *HNF4A* and *HNF1A* genes respectively, are associated with progressive β-cell dysfunction and typically respond very well to SUs, although insulin may eventually be required; a low-carbohydrate diet may also offer benefit [113, 114]. Lastly, MODY 5, related to *HNF1B* mutations, presents with pancreatic, renal, and genital malformations and generally requires insulin as first-line therapy [115]. Given the heterogeneity of MODY, the different therapeutic approaches among the various subtypes and the importance of genetic counseling, accurate diagnosis is essential. Genetic screening for MODY should be considered in individuals diagnosed at a young age who lack typical features of T1D and T2D, such as obesity or autoantibodies, and present with a strong family history of diabetes or stable hyperglycemia [116].

### Gestational diabetes mellitus

Gestational diabetes mellitus (GDM), identified between the 24th and 28th week of pregnancy, is initially managed through lifestyle modification, including individualized nutrition and physical activity [117, 118]. In many cases, this may be sufficient, but when pharmacologic treatment is needed, insulin is the preferred agent due to its safety in pregnancy [116]. Glyburide and metformin are not first-line choices, as they cross the placenta, but may be considered [118]. Similarly, observational data point to the potential safety of both GLP-1 RAs and SGLT2is during pregnancy; however, these findings are not conclusive and require further investigation [119].

### Pancreatogenic and other secondary forms of diabetes

Pancreatogenic diabetes results from damage to or loss of pancreatic tissue due to pancreatitis, trauma, neoplasia, or cystic fibrosis [120]. Patients with a history of pancreatic disease should be screened for diabetes, diagnosis being essential to initiate appropriate dietary management and pancreatic enzyme replacement if needed, and to tailor glycemic therapy while avoiding hypoglycemia [121]. Initial treatment typically involves metformin and/or insulin, but most patients eventually require insulin, while incretin-based therapies are generally avoided [116]. In some cases, such as following pancreatectomy, islet auto-transplantation may be considered [122]. In cystic fibrosis-related diabetes (CFRD), which arises from pancreatic exocrine insufficiency, early screening is crucial. Standard diabetes dietary recommendations do not apply to CFRD; instead, patients require high-calorie, high-fat, high-salt diets. Due to insulin deficiency, insulin therapy is the preferred treatment even at low doses, while oral agents are not recommended [123].

Post-transplant diabetes mellitus (PTDM) is a common complication following organ transplantation primarily driven by physiological stress and the use of immunosuppressive agents, particularly glucocorticoids and calcineurin inhibitors. During hospitalization, insulin therapy is typically initiated due to its effectiveness and flexibility in managing acute hyperglycemia [124]. After discharge, the choice of glucose-lowering therapy should be individualized based on the patient’s clinical status, hyperglycemia severity, and renal function [125].

In recent years, immune checkpoint inhibitors (ICIs) have also been associated with a distinct form of diabetes. ICI-induced diabetes mimics fulminant T1D, characterized by rapid onset, severe insulin deficiency, and a high risk of DKA. These patients require immediate and lifelong insulin therapy and close monitoring for metabolic decompensation. In contrast, for patients receiving alpelisib, a phosphatidylinositol-3-kinase (PI3K) inhibitor used in cancer treatment, insulin should be avoided as it may antagonize the drug’s anti-tumor effects by activating the PI3K/protein kinase B (Akt)/mammalian target of rapamycin (mTOR) pathway. Instead, glucose-lowering agents such as metformin, SGLT-2is, or pioglitazone are preferred in this setting, provided that they are clinically appropriate and well-tolerated [53].

## Management of dyslipidemia in diabetes

Dyslipidemia is a highly prevalent comorbidity in individuals with diabetes and a major contributor to the increased burden of CV morbidity and mortality [126]. In T2D, dyslipidemia predominantly results from IR, producing a characteristic atherogenic lipid profile comprising elevated triglyceride (TG) levels, reduced high-density lipoprotein cholesterol (HDL-C), and an increased concentration of small dense low-density lipoprotein (sdLDL) particles [127]. These sdLDL particles are highly atherogenic due to their greater endothelial permeability and susceptibility to oxidative modification [128]. In contrast, in T1D, dyslipidemia is more commonly linked to suboptimal glycemic control rather than IR [129]. Clinical assessment of dyslipidemia in diabetes begins with a fasting lipid profile before initiating any lipid-lowering therapy. Follow-up lipid panels are advised 4 to 12 weeks after starting or adjusting treatment, with annual monitoring thereafter [130, 131].

**Fig. 1:**
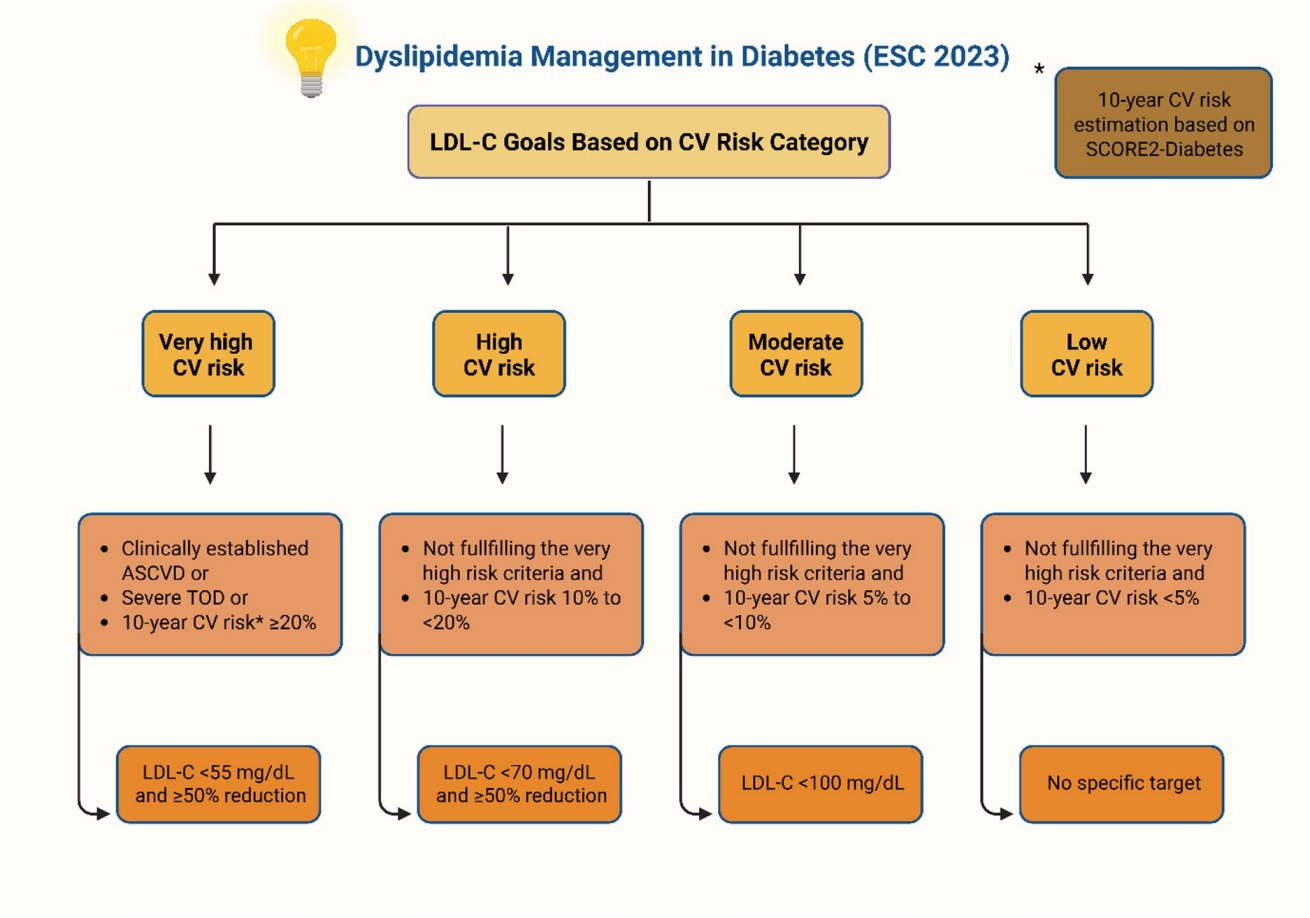
Therapeutic approach to dyslipidemia in patients with T2D according to the 2023 ESC guidelines. The figure illustrates LDL-C treatment goals based on CV risk categories (very high, high, moderate, and low). Risk classification is based on the presence of ASCVD, TOD, or estimated 10-year CV risk, the latter assessed using the SCORE2-Diabetes model (*). Abbreviations: T2D: type 2 diabetes; ASCVD: atherosclerotic cardiovascular disease; CV: cardiovascular; ESC: European Society of Cardiology; LDL-C: low-density lipoprotein cholesterol; TOD: target organ damage (TOD). Created in BioRender. Kounatidis, D. (2025) https://BioRender.com/47qsxwq

Across all major guidelines, CV risk stratification is central to determining the intensity of lipid-lowering therapy, with LDL-C serving as the primary therapeutic target. In the context of primary prevention, the ADA recommends high-intensity statin therapy for adults with T2D and at least one atherosclerotic cardiovascular disease (ASCVD) risk factor, aiming for LDL-C < 70 mg/dL. For adults aged ≥ 75 years, continuation of current statin therapy or initiation of moderate-intensity statins may be considered after individual risk-benefit evaluation. For secondary prevention, the LDL-C target is even stricter, set at < 55 mg/dL [131]. In Europe, the 2023 European Society of Cardiology (ESC) guidelines introduced an updated framework for risk stratification. For the first time, the presence of advanced target organ damage (TOD) and the SCORE2-Diabetes algorithm are utilized to estimate 10-year CV risk [132]. Based on these parameters and the presence or absence of established ASCVD, patients with T2D are classified into four CV risk categories, each with corresponding LDL-C targets, except for the low-risk group where limited data preclude defining a specific goal. For patients with T1D, recommendations vary primarily by age. For those under 40 years, treatment is advised if additional ASCVD risk factors, microvascular TOD, or a 10-year CV risk ≥ 10% are present. In individuals over 40, pharmacologic treatment is recommended regardless of ASCVD status [133]. Figure 1 illustrates the key components of the therapeutic approach of dyslipidemia in individuals with T2D, based on the 2023 ESC guidelines.

 Lifestyle modification remains vital to dyslipidemia management in diabetes. A Mediterranean diet, supported by strong evidence for CV effects, combined with regular physical activity, is highly recommended [134]. Statins are the first-line pharmacologic agents for lowering LDL-C. If LDL-C goals are not met, ezetimibe is typically added. In high- or very high-risk patients who do not achieve targets with maximally tolerated statin plus ezetimibe, PCSK9 inhibitors (e.g., evolocumab and alirocumab) or inclisiran (a small interfering RNA agent) may be employed. These agents also serve as options for patients with confirmed statin intolerance. Bempedoic acid offers another LDL-lowering option, though its availability is limited in Europe [131, 133]. In cases of hypertriglyceridemia, the initial focus is on optimizing lifestyle and improving glycemic control, as chronic hyperglycemia contributes significantly to elevated TG levels. For individuals with established ASCVD or at high CV risk, and fasting TG levels between 150-499 mg/dL, the addition of icosapent ethyl (2 g twice daily) to statin therapy is supported by the REDUCE-IT (Reduction of Cardiovascular Events with Icosapent Ethyl-Intervention Trial), in 2019, which showed a 25% relative risk reduction in CV events [135]. When TG levels exceed 500 mg/dL, fenofibrate is recommended to reduce the risk of acute pancreatitis. While elevated TG levels are linked to increased residual CV risk, evidence remains inconclusive as to whether lowering TGs directly reduces CV events [133].

## Diabetes treatment: a holistic approach

ASCVD remains the leading cause of death globally, with a notably higher prevalence in individuals with diabetes [136]. Hypertension, a key modifiable risk factor for ASCVD, should be managed to target blood pressure below 130/80 mmHg, although individualized targets may be appropriate in specific scenarios, including pregnancy [131]. First-line antihypertensive therapy in patients with diabetes includes agents proven to reduce CV events, primarily ACE inhibitors or ARBs, titrated and, if necessary, combined with other medications [137]. Lifestyle interventions are essential and include adherence to the DASH dietary pattern with reduced sodium intake, smoking cessation, and increased physical activity [138]. In patients with diabetes and established ASCVD or CKD, either an SGLT2i and/or a GLP-1RA should be incorporated into the treatment regimen. For individuals with HF, regardless of ejection fraction status, an SGLT2i with established CV benefit is preferred. If obesity is present, a GLP-1RA should be preferred [131]. In patients with T2D and CKD, finerenone may be added to ACE inhibitor or ARB therapy as it has demonstrated efficacy in improving CV outcomes and reducing hospitalization for HF [139]. Aspirin is recommended for secondary prevention; for primary prevention, its use is more selective and may be considered in individuals at high CV risk when the potential benefit outweighs bleeding risk [131, 140].

 Metabolic dysfunction-associated steatotic liver disease (MASLD) is the most common chronic liver disease, affecting approximately 65% of patients with T2D, with around 15% already presenting with advanced fibrosis:its prevalence may reach as much as 90% when T2D and obesity coexist [141]. All individuals with T2D should undergo fibrosis assessment using non-invasive tools such as the fibrosis-4 (FIB-4) index [142]. Weight loss remains the cornerstone of MASLD management and is best achieved through a Mediterranean dietary pattern, regular exercise, and avoidance of alcohol and smoking. A weight reduction of 5–7% is generally required to reverse steatosis and steatohepatitis, while improvement in fibrosis typically requires ≥10% weight loss [143]. GLP-1RAs, particularly semaglutide, have demonstrated efficacy in reducing hepatic steatosis and steatohepatitis, though they are not yet approved, at the usual antidiabetic doses, as a specific metabolic dysfunction-associated steatohepatitis (MASH) treatment due to insufficient evidence from phase 3 trials regarding their effect on fibrosis [144]. Pioglitazone has also shown beneficial effects on hepatic steatosis with potential anti-fibrotic effects, although its use is limited by the aforementioned side effects. Similarly, SGLT2is have demonstrated proven benefits, particularly in the early stages of fibrosis [145]. Lastly, tirzepatide has shown promise in a phase 2 trial, demonstrating both MASH resolution and fibrosis improvement by more than one stage, findings that warrant further validation [146]. In the MAESTRO-NASH trial (Metabolic Dysfunction-Associated Steatohepatitis Treatment with Resmetirom), published in February 2024, resmetirom, a selective thyroid hormone receptor-β (THR-β) agonist, demonstrated superiority over placebo in MASH resolution and fibrosis improvement by more than one grade. A few weeks later, in March 2024, the U.S. FDA approved resmetirom for the treatment of noncirrhotic MASH with moderate to advanced liver fibrosis (stages F2-F3) in the U.S. [147]. In addition to its effects on liver disease, resmetirom appears to have favorable effects on cardiovascular health too, as it improves lipid profiles and may help reduce cardiovascular risk [148]. Not until August 2025 was semaglutide 2.4 mg — a dose previously used for obesity rather than T2D — approved by the FDA for the treatment of MASH and moderate-to-advanced fibrosis (stages F2-F3), but not cirrhosis. The approval was based on histological improvements in both steatohepatitis and liver fibrosis, shown in the ESSENCE trial (Effect of Semaglutide in Subjects with Non-alcoholic steatohepatitis), in 2025 [149]. Despite the pharmacologic advances, lifestyle interventions, including diet, physical activity, and weight management, remain the foundation of MASLD treatment.

**Fig. 2:**
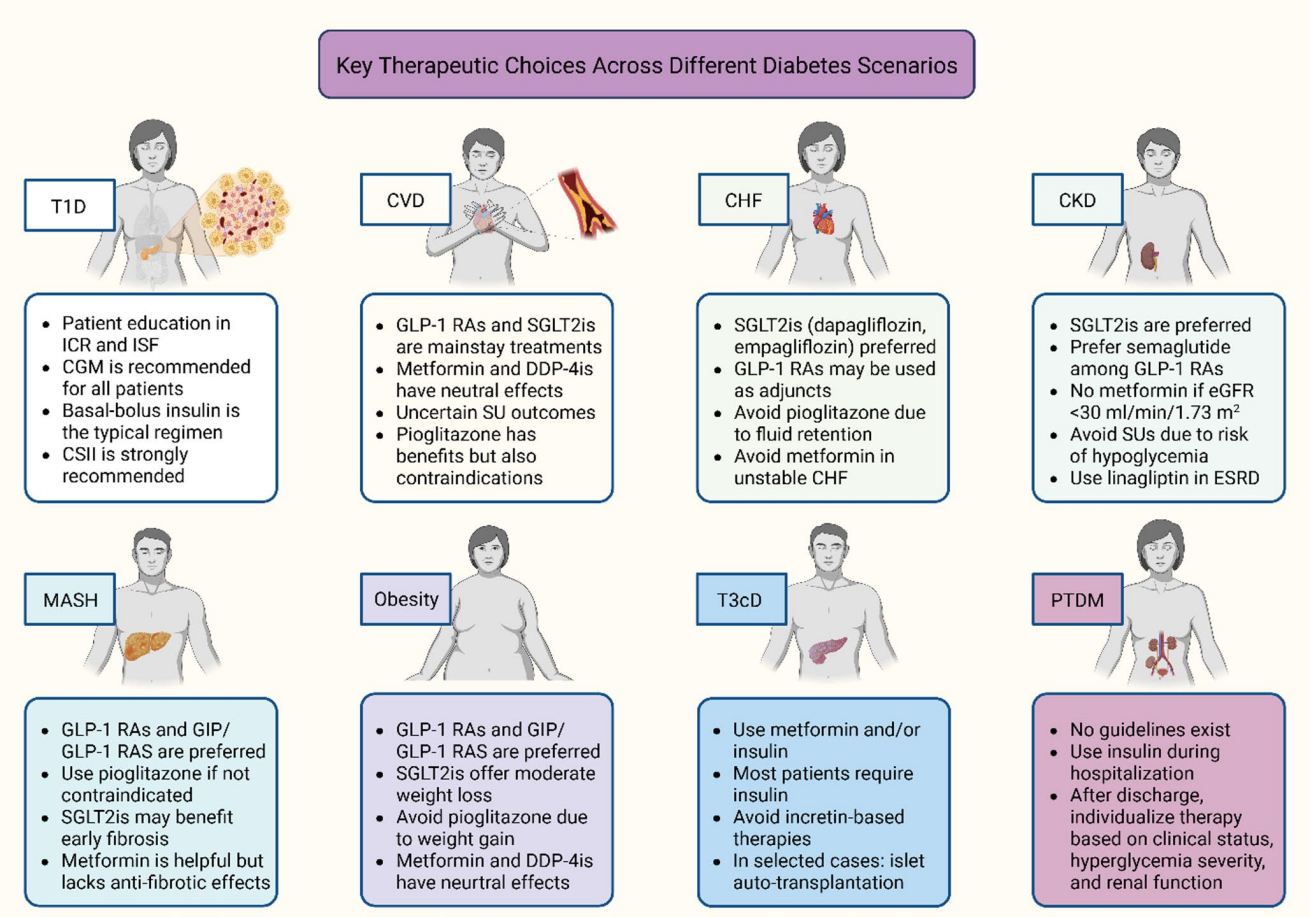
Key therapeutic choices across different diabetes scenarios. Abbreviations: CGM: continuous glucose monitoring; CHF: chronic heart failure; CKD: chronic kidney disease; CSII: continuous subcutaneous insulin infusion; CVD: cardiovascular disease; DDP-4is: dipeptidyl peptidase-4 inhibitors; eGFR: estimated glomerular filtration rate; ESRD: end-stage renal disease; GIP: glucose-dependent insulinotropic polypeptide; GLP-1 RA: glucagon-like peptide-1 receptor agonist; ICR: insulin-to-carbohydrate ratio; ISF: insulin sensitivity factor; MASH: metabolic dysfunction-associated steatohepatitis; PTDM: post-transplant diabetes mellitus; SGLT2is: sodium-glucose co-transporter-2 inhibitors; SU: sulfonylurea; T1D: type 1 diabetes; T3cD: type 3c diabetes. Created in BioRender. Kounatidis, D. (2025) https://BioRender.com/zlmmlxf

Routine monitoring of urinary albumin-to-creatinine ratio (UACR) and eGFR is standard in diabetes care as diabetes is the leading cause of CKD. ACE inhibitors or ARBs remain the preferred agents for managing albuminuria, while in diabetic kidney disease (DKD), SGLT2is are recommended for their ability to slow CKD progression and reduce CV and heart failure events, offering both renal and cardiac protection. GLP-1RAs may provide additional nephroprotective effects and are often co-administered with SGLT2is in DKD management. All medications must be dosed according to renal function [150]. Nutritional management should be tailored to individual needs, with a general recommendation of 0.8 g/kg/day of protein intake, adjusted upward in patients on dialysis, and reduced sodium intake [150]. Referral to a nephrologist is indicated when eGFR falls below 30 mL/min/1.73 m^2^, when UACR persistently increases, or when there is progressive decline in eGFR. Fig. 2 depicts eight distinct clinical scenarios of diabetes along with their corresponding therapeutic management strategies.

## ‘’The art of being a diabetologist’’

The pharmacologic armamentarium available to the modern diabetologist has expanded significantly in recent years, transforming the treatment landscape of T2D. Early initiation of the three novel glucose-lowering drug classes (SGLT2is, GLP-1 RAs, and tirzepatide) is strongly advocated to leverage their pleiotropic effects on cardiovascular and renal health. These agents not only enhance glycemic control but also directly address the underlying mechanisms driving diabetes-related complications, resulting in measurable improvements in both patient outcomes and QoL. A uniform treatment model is seldom adequate in diabetes management. The skilled clinician must tailor therapy, not solely through pharmacological choices, but also by fostering a therapeutic alliance with the patient. Effective care requires engaging patients in understanding the rationale behind each treatment, empowering them to adopt it meaningfully, and supporting long-term adherence.

 With at least eight distinct drug classes now available for T2D and multiple therapeutic combinations achieving comparable glycemic outcomes, individualization is no longer optional: it is essential. The role of the diabetologist extends beyond the control of glucose, HbA_1c_, lipids, albuminuria, and blood pressure. It involves crafting a personalized, sustainable plan that patients can commit to, minimizing side effects while lowering the risk of complications. Equally importantly, pharmacologic treatment must be grounded in a basis of lifestyle change. Strong evidence supports the metabolic and CV benefits of structured dietary patterns, particularly the Mediterranean and DASH diets. Regular physical activity, behavioral interventions, and psychological support frequently play integral roles, especially in individuals with obesity, emotional distress, and/or low treatment motivation. Symptoms related to hyperglycemia, such as polyuria, polydipsia, and increased hunger, must always raise clinical suspicion, even in previously well-controlled patients. While seemingly self-evident, such signs are often missed in high-volume public health settings where time constraints and competing priorities can dilute attention to subtle but important clinical cues. In these environments, the art of attentive listening and detailed clinical observation is more vital than ever.

 Overall, being a diabetologist demands more than technical proficiency. It calls for empathy, curiosity, vigilance, and a commitment to excellence in every clinical encounter. This is a transformative time in diabetes care, complex and demanding, but also deeply rewarding. Change begins not with sweeping declarations, but with the thoughtful, patient-centered decisions made one person at a time.

## Abbreviations

ACE: Angiotensin-converting enzyme
ADA: American Diabetes Association
AID: Automated insulin delivery
AMPK: AMP-activated protein kinase
ARBs: Angiotensin receptor blockers
ASCVD: Atherosclerotic cardiovascular disease
CBT: Cognitive behavioural therapy
CFRD: Cystic fibrosis-related diabetes
CHF: Chronic heart failure
CGM: Continuous glucose monitoring
CKD: Chronic kidney disease
CSII: Continuous subcutaneous insulin infusion
CV: Cardiovascular
CVD: Cardiovascular disease
CVOTs: Cardiovascular outcome trials
DASH: Dietary Approaches to Stop Hypertension
DKA: Diabetic ketoacidosis
DKD: Diabetic kidney disease
DPP: Diabetes Prevention Program
DPP-4is: Dipeptidyl peptidase-4 inhibitors
EASD: European Association for the Study of Diabetes
eGFR: Estimated glomerular filtration rate
ESC: European Society of Cardiology
ESRD: End-stage renal disease
FDA: Food and Drug Administration
FIB-4: Fibrosis-4 index
FPG: Fasting plasma glucose
GCK: Glucokinase
GDM: Gestational diabetes mellitus
GLP-1 RAs: Glucagon-like peptide-1 receptor agonists
GLP-1/GIP RA: Dual glucagon-like peptide-1/glucose-dependent insulinotropic polypeptide receptor agonist
HbA1c: Glycated hemoglobin A1c
HDL-c: High-density lipoprotein cholesterol
HF: Heart failure
HFpEF: Heart failure with preserved ejection fraction
HFrEF: Heart failure with reduced ejection fraction
HIIT: High-intensity interval training
ICIs: Immune checkpoint inhibitors
ICR: Insulin-to-carbohydrate ratio
IR: Insulin resistance
ISF: Insulin sensitivity factor
LADA: Latent autoimmune diabetes in adults
LDL-C: Low-density lipoprotein cholesterol
MACE: Major adverse cardiovascular events
MASLD: Metabolic dysfunction-associated steatotic liver disease
MASH: Metabolic dysfunction-associated steatohepatitis
MBS: Metabolic bariatric surgery
MDI: Multiple daily injections
MNT: Medical nutrition therapy
MODY: Maturity-onset diabetes of the young
mTOR: Mammalian target of rapamycin
NNS: Non-nutritive sweeteners
P13K: Phosphatidylinositol-3-kinase
PCT: Proximal convoluted tubule
PPAR-γ: Peroxisome proliferator-activated receptor gamma
PTDM: Post-transplant diabetes mellitus
QoL: Quality of life
RCTs: Randomized controlled trials
sLDL: Small dense low-density lipoprotein
SGLT2is: Sodium-glucose co-transporter-2 inhibitors
SUs: Sulfonylureas
T1D: Type 1 diabetes
T2D: Type 2 diabetes
T3cD: Type 3c diabetes
TG: Triglyceride
THR-β: Thyroid hormone receptor-β
TOD: Target organ damage
TZDs: Thiazolidinediones
UACR: Urinary albumin-to-creatinine ratio
UKPDS: UK Prospective Diabetes Study
U.S.: The United States of America

## Trial abbreviation

BRIGHT: Digital Therapeutic Based Investigation to Improve Glycemic Control in Patients With Type 2 Diabetes and Residual Hyperglycemia on Stable Medical Therapy
CANVAS: Canagliflozin Cardiovascular Assessment Study
CREDENCE: Canagliflozin and Renal Events in Diabetes with Established Nephropathy Clinical Evaluation
DAPA-CKD: Dapagliflozin and Prevention of Adverse Outcomes in Chronic Kidney Disease
DAPA-HF: Dapagliflozin and Prevention of Adverse Outcomes in Heart Failure
DECLARE-TIMI 58: Dapagliflozin Effect on Cardiovascular Events – Thrombolysis in Myocardial Infarction 58
DELIVER: Dapagliflozin Evaluation to Improve the Lives of Patients with Preserved Ejection Fraction Heart Failure
EMPA-KIDNEY: Study of Heart and Kidney Protection with Empagliflozin
EMPA-REG OUTCOME: Empagliflozin Cardiovascular Outcome Event Trial in Type 2 Diabetes Mellitus Patients – Removing Excess Glucose
EMPEROR-Preserved: Empagliflozin Outcome Trial in Patients With Chronic Heart Failure With Preserved Ejection Fraction
ESSENCE: Effect of Semaglutide in Subjects with Nonalcoholic Steatohepatitis
FLOW: Evaluate Renal Function with Semaglutide Once Weekly
MAESTRO-NASH: Metabolic Dysfunction-Associated Steatohepatitis Treatment with Resmetirom
LEADER: Liraglutide Effect and Action in Diabetes: Evaluation of Cardiovascular Outcome Results
REWIND: Researching Cardiovascular Events with a Weekly Incretin in Diabetes
SUSTAIN-6: Trial to Evaluate Cardiovascular and Other Long-term Outcomes with Semaglutide in Subjects with Type 2 Diabetes
SURPASS: Study of the Efficacy and Safety of Tirzepatide
SURPASS-2: Tirzepatide versus Semaglutide Once Weekly in Patients with Type 2 Diabetes
